# A Novel 3-Phytosterone-9α-Hydroxylase Oxygenation Component and Its Application in Bioconversion of 4-Androstene-3,17-Dione to 9α-Hydroxy-4-Androstene-3,17-Dione Coupling with A NADH Regeneration Formate Dehydrogenase

**DOI:** 10.3390/molecules24142534

**Published:** 2019-07-11

**Authors:** Xian Zhang, Manchi Zhu, Rumeng Han, Youxi Zhao, Kewei Chen, Kai Qian, Minglong Shao, Taowei Yang, Meijuan Xu, Jianzhong Xu, Zhiming Rao

**Affiliations:** 1 The Key Laboratory of Industrial Biotechnology, Ministry of Education, School of Biotechnology, Jiangnan University, 1800 Liu Avenue, Wuxi 214122, Jiangsu, China; 2 Biochemical Engineering College, Beijing Union University, Beijing 100023, China; 3 School of Food Science and Technology, Jiangnan University, 1800 Liu Avenue, Wuxi 214122, Jiangsu, China; 4 School of Medicine, Yichun University, Yichun 336000, Jiangxi, China

**Keywords:** 9α-Hydroxy-4-androstene-3,17-dione, 3-phytosterone-9α-hydroxylase, hydroxylation, NADH regeneration

## Abstract

9α-Hydroxy-4-androstene-3,17-dione (9-OH-AD) is one of the significant intermediates for the preparation of β-methasone, dexamethasone, and other steroids. In general, the key enzyme that enables the biotransformation of 4-androstene-3,17-dione (AD) to 9-OH-AD is 3-phytosterone-9α-hydroxylase (KSH), which consists of two components: a terminal oxygenase (KshA) and ferredoxin reductase (KshB). The reaction is carried out with the concomitant oxidation of NADH to NAD^+^. In this study, the more efficient 3-phytosterone-9α-hydroxylase oxygenase (KshC) from the *Mycobacterium* sp. strain VKM Ac-1817D was confirmed and compared with reported KshA. To evaluate the function of KshC on the bioconversion of AD to 9-OH-AD, the characterization of KshC and the compounded system of KshB, KshC, and NADH was constructed. The optimum ratio of KSH oxygenase to reductase content was 1.5:1. An NADH regeneration system was designed by introducing a formate dehydrogenase, further confirming that a more economical process for biological transformation from AD to 9-OH-AD was established. A total of 7.78 g of 9-OH-AD per liter was achieved through a fed-batch process with a 92.11% conversion rate (mol/mol). This enzyme-mediated hydroxylation method provides an environmentally friendly and economical strategy for the production of 9-OH-AD.

## 1. Introduction

Currently, steroids are widely used in anti-tumor, anti-inflammation, anti-microbial, anti-viral, and antifungal drugs among others, and they are the most important drugs apart from antibiotics. Steroid compounds usually have a series of unique physiological functions that mainly result from differences in the position of the substituent or double bond or in the configuration [1]. Mycobacteria have the advantage of using natural sterols or other steroids as the only carbon and energy sources. Previous research has shown that by interrupting mycobacterial pathways, some important intermediates can be accumulated, such as 4-androstene-3,17-dione (AD), 1,4-androstene-3,17-dione (ADD), and 9α-hydroxy-4-androstene-3,17-dione (9-OH-AD) and so on [2], which can then be used as precursors in producing steroid hormones. AD and ADD can be used commercially to produce glucocorticoids, oral contraceptives, and other steroid drugs. 9-OH-AD can be used to produce glucocorticoid drugs by replacing the 9α-hydroxyl group with a halogen group; thus, 9-OH-AD is an important precursor for the preparation of steroid drugs [3,4,5].

The hydroxylation of steroids is widespread in microorganisms. The effects and mechanisms of steroid drugs with different substituent positions or different conformations, including those with 9-α, 11-α, 11-β, 15-α, and 16-α hydroxylation, also vary; 9-OH-AD is hydroxylated from AD. The specificity of the hydroxylation reaction depends on the catalytic enzyme used in the process. 3-Phytosterone-9α-hydroxylase (KSH), which was used in this study, is a key microbial steroid metabolic enzyme that widely exists in microorganisms, such as *Rhodococcus* [6], *Mycobacterium* [7], *Nocardia* [8], and *Arthrobacter* [9]. KSH is composed of two different components, including KSH oxygenase (KshA) and KSH reductase (KshB), which are encoded by the genes *kshA* and *kshB*, respectively. Petrusma et al. confirmed that the function of KSH in *Rhodococcus erythropolis* SQ1 must be completed by two components, both of which are necessary [6]. The reductase component KshB, which contains FAD, an NAD-binding domain and a [2Fe-2S] redox center, is responsible for transferring the reductive force from NADH to KshA. The hydroxylation reaction and the binding of O_2_ with the substrate occurs in the non-heme Fe^2+^-binding region of KshA. In addition, KshB, functioning as a reductive force, can also play a role in other reactions in an organism [10].

At present, most studies have been focused on the production of 9-OH-AD by bacterial fermentation. An increasing number of microorganisms have been screened for the ability to modify steroid molecules to promote the development of steroid drugs [11,12,13,14]. However, there must be some rate-limiting steps and rate-limiting enzymes in the sterol degradation pathway. In addition, because 3-ketosteroid-Δ1-dehydrogenase (Ksdd) can catalyze the conversion of AD to ADD, the accumulation of 9-OH-AD is hindered, leading to a low conversion rate with a longer fermentation period, greatly reducing the production efficiency [15,16,17,18]. For example, Donova et al. used the mutant *Mycobacterium* sp. 2-4 M strain to produce 9-OH-AD, and the conversion rate (mol/mol) was approximately 50% [19]. The KshA, KshB and glucose dehydrogenase (GDH) of *Mycobacterium neoaurum* JC-12 were coexpressed in *Bacillus subtilis* 168, and the effective conversion of AD to 9-OH-AD was realized [20]. In mycobacteria, knocking out the gene *ksdd* and overexpressing KshA leads to the accumulation of 9-OH-AD (approximately 6.78 to 7.33 g/L), but the fermentation period exceeds 150 h [21]. *Mycobacterium* sp. VKM Ac-1817D (GenBank accession number: CP009914) can use phytosterol as a substrate to produce 9-OH-AD by bioconversion. Although the product contains 20% by-products such as ADD and 9-OH-ADD [22], this strain of bacterium produces relatively high yields of 9-OH-AD. Therefore, this strain was selected as the source of KSH in this study. From searches on the genome of *Mycobacterium* sp. VKM Ac-1817D, three genes *kshA*, *kshB*, and *ksh* annotated as KSH oxygenase, KSH reductase, KSH, respectively, were found. To make it easier to distinguish these genes, we renamed *ksh* as *kshC*, and the coding enzymes of the three genes were KshA, KshB, and KshC, respectively.

**Figure 1:**
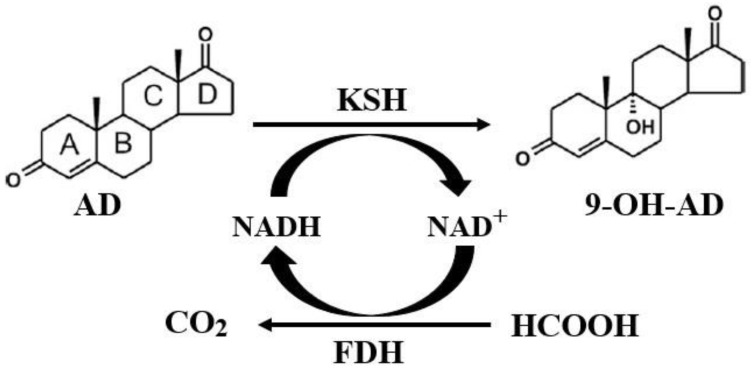
Coupled reaction of hydroxylation and NADH regeneration. KSH: 3-phytosterone-9α-hydroxylase. FDH: formate dehydrogenase. AD: 4-androstene-3,17-dione. 9-OH-AD: 9α-hydroxy-4-androstene-3,17-dione.

Given that the reductive force of the conversion process, NADH, must be restored, this study constructed a coupled system formed by hydroxylation catalyzed by KSH and coenzyme regeneration catalyzed by formate dehydrogenase (FDH) (Figure 1), thus greatly reducing the transformation period, improving the conversion rate, and reducing the cost, resulting in a more economical and practical system.

NADH regeneration strategies, such as enzymatic, biological, electrochemical, chemical, and photochemical methods, have been developed [23]. FDH, which catalyzes the oxidation of formate to carbon dioxide, is an enzyme that is capable of regenerating NADH. The advantages of using FDH in the coenzyme regeneration system include the following: Formic acid is relatively cheap as a substrate; little damage occurs to the enzyme; as a gas, CO_2_ can be separated directly without inhibiting product formation [24]; the pH application range is relatively wide; and FDH can play a highly effective and stable catalytic role in the range of pH 6 to 9 [25]. Furthermore, the reaction is irreversible and can obviously improve the recovery of the product. Multiple reports describe FDH double enzyme coupling, which is widely used in oxidation-reduction reactions. In 2002, Matsuyama et al. expressed alcohol dehydrogenase and FDH in *Escherichia coli* and used the whole cells to transform acetic acid ethyl acetate into (S)-4-chloro-3-hydroxybutyrate via an asymmetric reduction [26]. Galkin determined that more than one amino acid dehydrogenase in *E. coli* was expressed, including leucine dehydrogenase and phenylalanine dehydrogenase, and these amino acid dehydrogenases, along with formic acid dehydrogenase, produce L-alanine [27]. We propose an alternative strategy for the production of 9-OH-AD by KSH coupled with the regeneration of NADH by FDH.

## 2. Results and Discussion

### 2.1. Heterologous Expression and Identification of the KshA, KshB, and KshC Proteins in E. coli

By searching of the genome of *Mycobacterium* sp. VKM Ac-1817D (GenBank accession number: CP009914), three genes*—shA* (encoding the 3-ketosteroid-9-alpha-hydroxylase oxygenase component), *kshB* (encoding the 3-ketosteroid-9-alpha-hydroxylase oxygenase component), and *ksh* (named *kshC* in this work; it has an unknown function and was annotated as encoding the 3-ketosteroid-9-alpha-hydroxylase)—were found. The KSH genes were synthesized according to the codon preferences of *E. coli*. The amplified primers used were designed and synthesized based on the full-length of these *ksh* sequences; therefore, *kshA*, *kshB,* and *kshC* were recovered as 1164-bp, 1059-bp, and 1164-bp fragments, respectively. After the ligation of *ksh* and pet-28a(+), as well as the transformation of the plasmids into *E. coli* BL21 (DE3), the recombinant bacteria, BL21 (DE3)/pet-28a(+)-*ksh,* were randomly selected from the kanamycin-resistant agar-plates, and the plasmids were extracted and confirmed by restriction enzyme digestion using *Bam*H I and *Hind* III for single/double cut. As shown in Supplementary File 1: Figure S1, the double enzyme digestion produced a band that is consistent with the size of the target gene; therefore, it can be preliminarily concluded that each recombinant plasmid contained a *ksh.* The target gene was successfully ligated to pet-28a(+) and transformed into *E. coli* BL21 (DE3).

**Figure 2:**
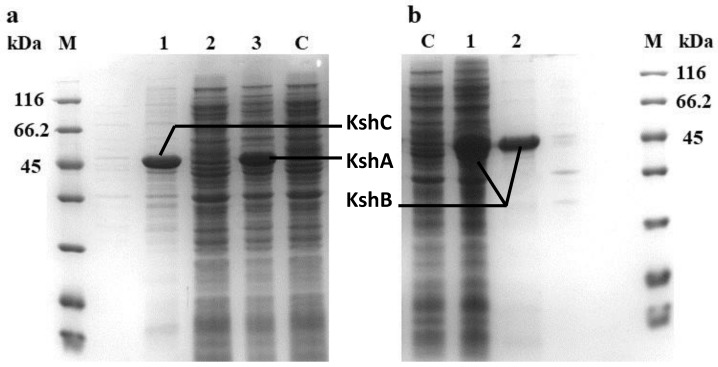
SDS-PAGE analysis of proteins in the parent bacteria and recombinant bacteria. (**a**) M: protein marker (kDa); C: supernatant of *E. coli* BL21/pET-28a; 1: sediment of *E. coli* BL21/pET-28a-*kshC* 2: supernatant of *E. coli* BL21/pET-28a-*kshC*; 3: supernatant of *E. coli* BL21/pET-28a-*kshA*. (**b**) M: protein marker (kDa); C: supernatant of *E. coli* BL21/pET-28a; 1: supernatant of *E. coli* BL21/pET-28a-*kshB*; 2: purified supernatant of *E. coli* BL21/pET-28a-*kshB.*

The engineered strains BL21 (DE3)/pet-28a(+)-*kshA*, BL21 (DE3)/pet-28a(+)-*kshB*, and BL21 (DE3)/pet-28a(+)-*kshC* were induced by the methods described in the Materials and Methods section, and the expression of the target proteins was then detected by SDS-PAGE. The results showed that the expression levels of the KshA and KshB proteins were higher than 30% of the total protein, and the expressed protein was mainly soluble. A clear band can be seen near 45 kDa, which is consistent with the expected size (KshA at 44.23 kDa and KshB at 37.48 kDa). However, in the present work, the SDS-PAGE results showed that almost all of the KshC protein produced was in the insoluble fraction and was considered to be inclusion bodies (Figure 2), an aggregated form of inactive proteins.

**Figure 3:**
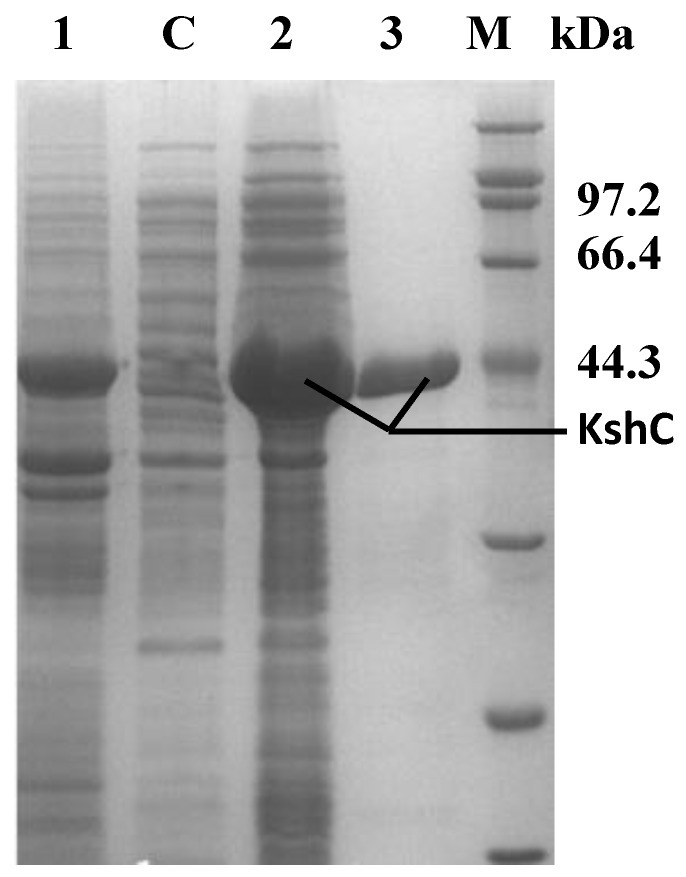
SDS-PAGE analysis of proteins in the parent bacteria and recombinant bacteria. M: protein marker (kDa); C: supernatant of *E. coli* BL21/pEtDuet-1; 1: sediment of *E. coli* BL21/pEtDuet-1-*kshC* 2: supernatant of *E. coli* BL21/pEtDuet-1-*kshC*; 3: purified supernatant of *E. coli* BL21/pEtDuet-1-*kshC*.

It is imperative to reduce the formation of insoluble substances and increase the yield of soluble KshC because of the severe reduction in the production of soluble KshC and the inhibition of KshC activity by inclusion bodies. In this study, the problem of expressing soluble KshC was solved by means of changing the expression vector. The *kshC* gene was ligated to the pEtDuet-1 plasmid to construct the recombinant bacterium *E. coli* BL21 (DE3)/pEtDuet-1-*kshC*; this bacterium successfully expressed KshC after induction culture and showed mainly soluble expression (Figure 3).

The size of both *kshC* and *kshA* is 1164 bp, and the sequence identity of these two genes is 67.25%. The identity between *kshC* and *kshB* is only 34.91%; therefore, it was speculated that the KshC protein functions as an oxygenase. To confirm this conjecture, the crude KshC enzyme solution was mixed with a crude KshA or KshB enzyme solution and then added to the transformation system used for the biotransformation of AD. The results showed that KshC and KshB catalyzed the conversion of AD to 9-OH-AD; conversely, the mixture of KshC and KshA could not catalyze the reaction, proving that KshC is indeed a KSH oxygenase component. More importantly, compared with KshA and KshB, KshC and KshB had higher catalytic efficiency and 9-OH-AD productivity.

### 2.2. Purification and Characterization of the KshB Protein

**Figure 4:**
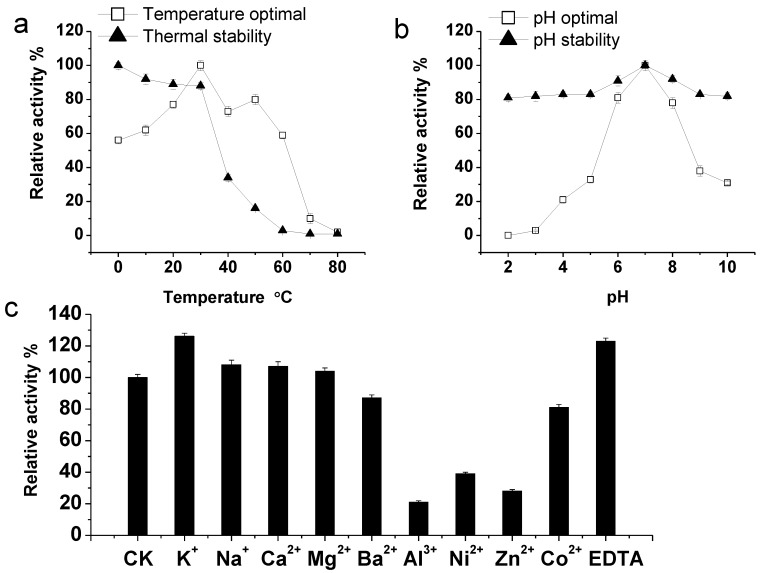
Enzymatic characterizations of KshB. (**a**) Temperature optimum and thermal stability of KshB. (**b**) pH optimum and pH stability of KshB. (**c**) Effect of metal ions and EDTA on KshB activity.

After the ultrasonic fragmentation of bacteria obtained from the induction culture, a crude enzyme solution was obtained by collecting the supernatant after high-speed frozen centrifugation. KshB was purified using a Ni affinity chromatography column. The purification of the target protein was validated by SDS-PAGE and the results are shown in Figure 3b. There is a specific band near 45 kDa that corresponds to the relative theoretical molecular weight calculated for the target protein (approximately 37.48 kDa), and the activity of KshB was 11.73 U/mg. Consistent to previous findings, harboring a flavin and a plant-type iron-sulfur cluster [28], the purified KshB presented an orange color, and NADH was a strict electron donor when the reductase activity of KshB was assayed with DCPIP [6]. The optimal temperature for KshB was 30 °C, and the enzyme was relatively stable under 30 °C (Figure 4a). The optimal pH for KshB was 7.0 (Figure 4b). The figure indicates that the purified KshB protein showed more stable activity in a wide pH range; thus, the purified enzyme was less sensitive to the pH than to temperature. Regarding metal ions, KshB was inhibited by Ba^2+^, Al^3+^, Ni^2+^, Zn^2+^, and Co^2+^; conversely, K^+^, Na^+^, Ca^2+^, and EDTA had obvious activation effects on KshB (Figure 4c).

### 2.3. Optimization of the Terminal Oxygenase and Ferredoxin Reductase Ratio in an Enzyme-Liquid Transformation System

**Figure 5:**
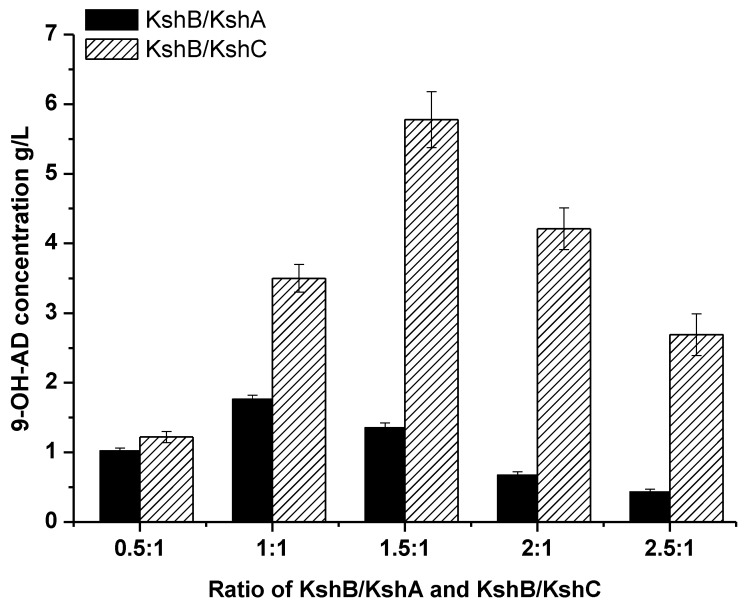
Optimization of the oxygenase and reductase ratio of KSH (KshB/KshA and KshB/KshC). The reaction was performed in 50 mM Tris-HCl buffer (pH 7.0) containing 105 mM NADH and 20 mM AD, and was assayed at 30 °C. All assays were performed in triplicate, and standard deviations of the biological replicates are shown.

According to the analysis of the temperature stability of the enzymatic properties of KshB, the enzyme is relatively stable at 30 °C or lower; therefore, the temperature was controlled at 30 °C. The role of an oxygenase requires an adequate amount of oxygen by increasing the agitation speed of the catalytic process to increase the oxygen content. Under such an optimized condition, the conversion of AD to 9-OH-AD by KSH can be more effective. The optimal ratio (crude enzyme) of KshB to KshC content was 1.5:1 while that of KshB to KshA was 1:1 (Figure 5) because the activity of an oxygenase is more stable in a reducing environment or the oxygenase would be easily inactivated. Thus, more KshB is needed to provide a reducing environment for KshC. However, the activity of KshA is, to some degree, lower than that of KshC; therefore, more KshA is required. The optimal ratio of KshB to KshC of 1.5:1 was employed in the following experiments. Under optimal conditions, KshC with KshB can produce approximately 5.9 g/L of 9-OH-AD in 6 h with a 97.5% conversion rate (mol/mol) in contrast to 1.8 g/L by KshA with KshB.

### 2.4. Coupled Reaction of Hydroxylation and NADH Regeneration and the Optimization of the Transformation System

The regeneration of NADH contributes to a more economical reaction because the conversion process from AD to 9-OD-AD needs NADH as the electron acceptor. Thus, KSH from *Mycobacterium* sp. VKM Ac-1817D and FDH from *Candida boidinii* were coupled in this work by separately overexpressing them in *E. coli* BL21 (DE3). The crude enzyme activities of the recombinants used in the NADH regeneration system are listed in Table 1.

**Figure 6:**
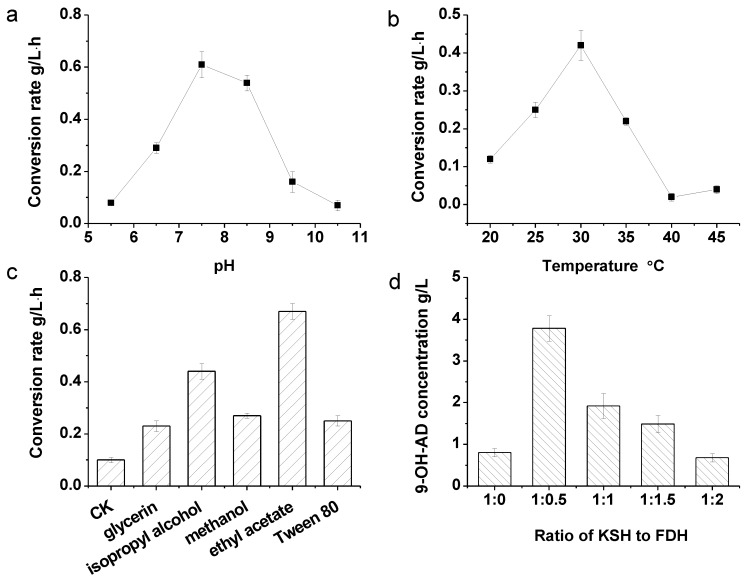
Optimization of the transformation system. (**a**) Effect of reaction pH on 9-OH-AD productivity (represented as 9-OH-AD concentration per liter transfer solution per hour) by the enzymatic system. (**b**) Effect of reaction temperature on 9-OH-AD productivity by the enzymatic system. (**c**) Effect of co-solvents on 9-OH-AD productivity by the enzymatic system. (**d**) Effect of KSH to FDH ration on 9-OH-AD productivity by the enzymatic system. When optimizing the effect of pH, temperature, and co-solvents on the enzymatic system, FDH was supplied excessively. All assays were performed in triplicate, and standard deviations of the biological replicates are shown.

In the mixed multi-enzyme catalyst, the conditions required by each enzyme should be taken into account, such as temperature and pH conditions. The optimal temperature for KshB activity is 30 °C, and the pH optimal of KshB is 7.0. As reported in a previous work, the optimal pH and temperature conditions of FDH used in this work were approximately from pH 6.5 to 8.5 and 37 °C, respectively [29]. To make the enzymes play more comparative roles in the coupled reaction, the biocatalytic conditions should be optimized. As shown in Figure 6, the optimal pH, temperature, solvents, and ratio of KSH to FDH of the reaction were investigated. The results showed that pH 7.5 (Figure 6a) and 30 °C (Figure 6b) were optimal and were thus selected and employed in the following experiments.

Considering that the substrate is insoluble in water, we tested the auxiliary effects of many solvents, including glycerin, isopropyl alcohol, methanol, ethyl acetate, and Tween 80. The concentrations of co-solvents were selected at 2% (v/v), as reported in our previous work [30]. Ethyl acetate was considered to be the best co-solvent (Figure 6c). It was inferred that AD dissolved in ethyl acetate may be more homogeneous in a water phase transformation system. Another probable reason is that ethyl acetate leads to only minor damage in enzyme activity.

The KSH to FDH ratio was further investigated while the ratio of KshB to KshC was maintained at 1.5:1 (Figure 6d). It can be concluded that 1:0.5 is the favorable ratio of KSH to FDH activity in a 6-h catalysis, and 3.88 g/L of 9-OH-AD was produced, which is 4.17-fold higher than that produced when no FDH was added (0.93 g/L). However, further increases in the proportion of FDH activity failed to improve the yield of 9-OH-AD. Therefore, we used the ratio of 1:0.5 (KSH to FDH activity) in subsequent experiments.

### 2.5. Fed-Batch Conversion under Optimal Conditions for the Biocatalysis of 9-OH-AD

**Figure 7:**
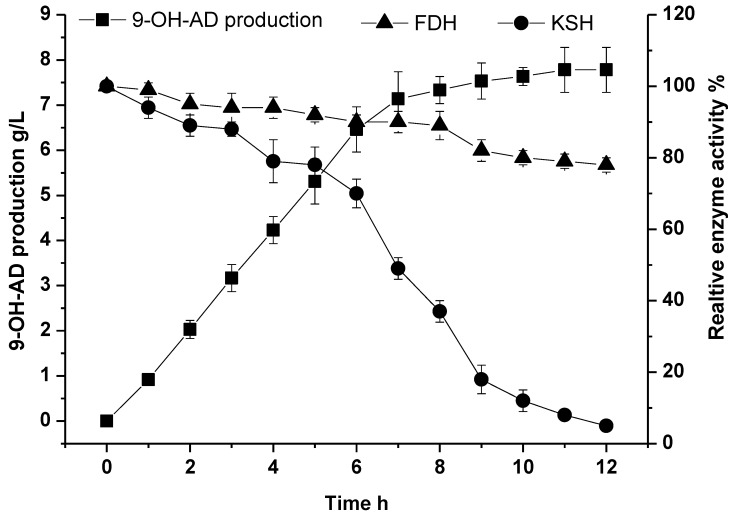
Time courses of fed-batch bioconversion from AD to 9-OD-AD by KSH and FDH. The production of 9-OD-AD and enzyme activities of KSH and FDH were assayed. All assays were performed in triplicate, and standard deviations of the biological replicates are shown.

Conversion strategies can be applied to improve the concentration of desired product. Considering the low solubility of AD and its inhibition effect to enzymes, we employed fed-batch strategy by feeding 1.0 g/L of AD into the conversion system per hour. As shown in Figure 7, 7.14 g/L of 9-OH-AD was transformed from AD over the first seven batches. However, the total 9-OH-AD production grew slowly after the eighth batch, because high concentration of 9-OH-AD inhibits KSH activity (Supplementary File 2: Figure S2). Finally, after 11 h of bioconversion, a total of 8 g/L of AD was transformed into 7.78 g/L of 9-OH-AD with a conversion rate of 92.11% (mol/mol). 

## 3. Materials and Methods

### 3.1. Chemicals, Primers, Plasmids, and Bacterial Strains

The standard samples of AD and 9-OH-AD were purchased from Sigma-Aldrich Co. LLC (Shanghai, China). The yeast extract and the tryptone were purchased from Oxoid Ltd. (Basingstoke Hampshire, England). All other chemicals were obtained from Sangon Biotech., Ltd. (Shanghai, China) and were of analytical grade. The primers, plasmids, and bacterial strains used in this study are listed in Table 2.

### 3.2. Construction of Recombinant Plasmids and Strains

The *Mycobacterium* sp. strain VKM Ac-1817D *ksh* genes *kshA*, *kshB*, and *kshC* encoding KshA, KshB, and KshC were codon-optimized and synthesized by Sangon Biotech., Ltd. (Codon optimized sequences were listed in Supplementary File 5). Using primer pairs P1/P2 and P3/P4, *kshA* and *kshB* were amplified by PCR for further expression KshA and KshB on plasmid pET-28a(+), respectively. P5/P6 and P7/P6 were used to amplify the *kshC* gene by PCR for further expression of KshC on plasmids pET-28a(+) and pEtDuet-1, respectively. The over-expressed enzymes were fused with His-Tags upstream of their amino acids sequences. The PCR protocol has been described previously by Zhang et al. [31]. Plasmids pET-28a(+)-*kshA*, pET-28a(+)-*kshB*, pET-28a(+)-*kshC* and pEtDuet-1-*kshC* were constructed by ligation of the gene fragments and the linearized plasmids after digestion using the relevant restriction enzymes. The plasmids were then transformed into *E. coli* BL21 (DE3) cells using the calcium chloride method. Modified FDH was obtained from our laboratory [29]. Luria-Bertani (LB) medium containing peptone (10 g/L), yeast extract (5 g/L), and NaCl (10 g/L) was used for strain cultivation, and kanamycin (Km, 100 mg/mL) or ampicillin (Amp, 200 mg/mL) was added when needed.

### 3.3. Expression and Purification of Enzymes

A single colony of recombinant strains was inoculated into LB medium containing corresponding antibiotics and incubated on a rotary shaker (180 rpm, 37 °C) to an optical density at 600 nm of about 0.8. Isopropyl β-D-1-thiogalactopyranoside (IPTG, 80 mg/mL) was added to LB medium for inducing the expression of proteins, and the culture was then grown at 25 °C for 12 h on a rotary shaker at 180 rpm. The induced cells were centrifuged, washed three times by 50 mM PB buffer (Na_2_HPO_4_-NaH_2_PO_4_, pH 7.5), and then re-suspended in PB buffer. The cells were sonicated and centrifuged to obtain a crude enzyme solution. The crude enzyme solution was purified using a Ni-ATA sepharose prepacked column HisTrap HP (GE Healthcare, Uppsala, Sweden). The pooled fractions were then loaded on a SuperdexTM 200 (10/300GL), equilibrated with 20mM Tris-HCl (pH 8.0) and 150 mM NaCl, using an ÄKTA Protein Purifier system (Pharmacia, Uppsala, Sweden). According to the different chelation degree of His-Tag and nickel ion, the protein was linearly eluted by an elution buffer with different concentrations of imidazole, and the purified enzyme solutions were checked by SDS-PAGE.

### 3.4. Assay of KshB, KSH, and FDH Activities

The KSH and FDH crude enzymes were prepared following the protocols described by Qi et al. [32]. For assaying the enzyme activity of KshB, the system included the following: 0.1 mM DCPIP, 50 mM Tris-HCl buffer (pH 7.0), and 0.25 mM NADH; the role of coenzyme NADH is to provide electrons as an electron donor via the reductase activity of KshB, and DCPIP serves as the electron acceptor. The reaction is chromogenic, and DCPIP is soluble in the Tris-HCl buffer solution after turning dark blue. Once the original state is restored, the color of DCPIP fades and becomes colorless. Upon adding a certain amount of pure KshB enzyme or thick enzyme sample fluid, the change in the OD_340_ absorbance value was determined.

The system used to measure KSH enzyme activity included 105 mM NADH, 200 mM AD substrate (soluble in 100% isopropyl alcohol), 50 mM Tris-HCl buffer (pH 7.0), and KSH enzyme (including an oxygenation component and a reduction component). After incorporating NADH, Tris-HCL buffer, and KSH enzyme, a certain amount of AD substrate in isopropyl alcohol was added and the change in the OD_340_ absorbance value was determined; a unit of enzyme activity was defined as the amount of enzyme required to catalyze the conversion of 1 µmol of NADH to NAD^+^ within 1 min [6]. FDH activity was assayed as described by Berrios-Rivera et al. [33]. The protein concentrations were determined using the Bradford Protein Assay Kit.

### 3.5. Characterization of KshB

For the temperature optimum assay of KshB, the reactions were conducted at different temperatures ranging from 0 to 80 °C with a gradient of 10 °C. For the KshB thermal stability assay, the enzyme was pre-incubated at different temperatures for 5 h and then assayed at 30 °C. The following buffers were used to investigate the pH dependence of KshB: 0.1M HAc-NaAc (pH 2–4), Na_2_HPO_4_-NaH_2_PO_4_ (pH 5–7), and Tris-HCl (pH 8–10). The pH stability of KshB was investigated using the standard KshB assay after it had been pre-incubated at different pH buffers for 5 h. Metal ions and EDTA were investigated to verify their effects on KshB activity. After the KshB is dialyzed with 0.1 M Tris-HCl buffer, 0.1 mM metal ions and EDTA were added separately into the enzyme samples, which were then assayed under the standard condition.

### 3.6. Optimization of Conversion Conditions

The ratios of KSH reductase and oxygenase were optimized to achieve high conversion efficiency. The assays were performed in 50 mM Tris-HCl buffer (pH 7.0) containing 105 mM NADH and 20 mM AD at 30 °C, and the enzyme activities of the two components (KshB/KshA and KshB/KshC) were arranged as 0.5:1, 1:1, 1.5:1, 2:1, and 2.5:1, respectively.

When coupling KSH and FDH to construct a NADH regeneration system, 1 M ammonium formate was added as the co-substrate and the initial NADH concentration was 0.32 mM. The pH and temperature optimal of the NADH regeneration system were also optimized. The pH optimal was assayed under different pH conditions ranging from pH 5.5 to 10.5, and the temperature dependence was assayed at different temperatures ranging from 20 to 45 °C with a gradient of 5 °C. The effects of co-solvents on 9-OH-AD productivity by the enzymatic system were also investigated. Glycerin 2% (*v/v*), isopropyl alcohol, methanol, ethyl acetate, and Tween 80 were added separately as co-solvents into the reaction solution. The ratio of KSH/FDH was optimized with their comparative activities at 1:0, 1:0.5, 1:1, 1:1.5, and 1:2, respectively.

### 3.7. Production of 9-OH-AD by Fed-Batch Conversion

When applying the fed-batch conversion strategy, KSH and FDH were added according to the optimal ratio of the enzyme activities. The enzymatic reaction was conducted in Tris-HCl buffer (50 mM) and was maintained at pH 7.5 on a magnetic stirrer at 30 °C and 160 rpm. The initial concentration of ammonium formate and NADH was 1 M and 0.32 mM, respectively. AD at a concentration of 1.0 g/L was fed into the reaction solution per hour.

### 3.8. Analysis of Products

One milliliter of reaction sample was extracted with 4 mL of ethyl acetate and centrifuged to obtain supernatant for future determination. The concentrations of AD and 9-OH-AD were assayed via HPLC with a UV detector at 254 nm using a C18 column (Diamonsil C18, 5-μm particles, 250 mm × 4.6 mm). The composition of methanol and water in the mobile phase was 7/3 (v/v). The column temperature was 30 °C and the flow rate was set as 1 mL/min [34]. The conversion rate was calculated using the following equation: Conversion rate = m_9-OH-AD_/m_AD_, where m_9-OH-AD_ is the titer of 9-OH-AD produced from AD and m_AD_ is the titer of the substrate AD. The results of the HPLC samples are shown in Supplementary File 3: Figure S3.

After conversion of AD in the reaction solution, a mass of transformation products was extracted with ethyl acetate and then re-dissolved in methanol. The transformation products were separated by silica gel column chromatography; the eluent was petroleum ether/ethyl acetate system, the volume ratio of petroleum ether and ethyl acetate is 6:4, and the flow rate was 1–2 mL/min. The eluent was concentrated and vacuum treated and then analyzed by TLC to obtain the purified product. The structures of the purified product were elucidated by Avance III 400 MHz nuclear magnetic resonance (NMR) (1HNMR USES CD4O as a solvent). The NMR result of 9-OH AD is shown in Supplementary File 4: Figure S4.

## 4. Conclusions

In this work, the *ksh* genes from *Mycobacterium sp*. strain VKM Ac-1817D were cloned and overexpressed in *E. coli* BL21 (DE3). The function of a novel 3-phytosterone-9α-hydroxylase oxygenation component (KshC) was identified to have higher catalytic efficiency than the known oxygenation component (KshA). Then, an enzyme-mediated hydroxylation system for 9-OH-AD production was designed and successfully developed by applying KSH (KshB and KshC) coupled with NADH regeneration via FDH. Through optimization of the conversion conditions, type of solvent, ratio of enzymes, and applying of a fed-batch strategy, the conversion rate of AD to 9-OH-AD was greatly improved. This study provides a promising bioconversion method for 9-OH-AD production.

## Figures and Tables

**Table 1:** Assays of KshB, KSH, and FDH activity in various strains.

Strains	Enzymes	Substrate	Activity (U/mL)	Total Protein (mg/mL)	Specific Activity (U/mg protein)
*E. coli* BL21 (DE3)	KshB	DCPIP	0.64 ± 0.03	11.71 ± 0.15	0.05 ± 0.002
*E. coli* BL21 (DE3)	FDH	NADH	n.d.	12.21 ± 0.18	n.d.
*E. coli* BL21 (DE3)	KSH	AD	n.d.	2.095 ± 0.07	n.d.
BL21 (DE3)/pET-28a(+)-*kshB*	KshB	DCPIP	33.32 ± 0.12	11.25 ± 0.23	2.96 ± 0.12
BL21 (DE3)/pET-28a(+)-*fdh*	FDH	NADH	0.48 ± 0.02	12.13 ± 0.12	0.04 ± 0.002
BL21(DE3)/pEtDuet-1-*kshC*	KSH	AD	0.91 ± 0.06	2.07 ± 0.08	0.43 ± 0.02

n.d., not detected enzyme activity; All assays were performed in triplicate, and standard deviations of the biological replicates are shown.

**Table 2:** Bacterial strains, plasmids, and primers used.

Strains/Plasmids/Primers	Characteristics	Source
**Strains**		
*Escherichia coli*		
*E. coli* BL21(DE3)	*F- dcm ompT hsdS (rB- mB-) gal λ(DE3)*	Invitrogen
BL21 (DE3)/pET-28a(+)	BL21 (DE3) containing pET-28a(+) (Km^R^)	This study
BL21 (DE3)/pET-28a(+)-*kshA*	BL21 (DE3) containing pET-28a(+)-*kshA* (Km^R^)	This study
BL21 (DE3)/pET-28a(+)-*kshB*	BL21 (DE3) containing pET-28a(+)-*kshB* (Km^R^)	This study
BL21 (DE3)/pET-28a(+)-*kshC*	BL21 (DE3) containing pET-28a(+)-*kshC* (Km^R^)	This study
BL21 (DE3)/pET-28a(+)-*fdh*	BL21 (DE3) containing pET-28a(+)-*fdh* (Km^R^)	This study
BL21 (DE3)/pEtDuet-1	BL21 (DE3) containing pEtDuet-1 (Amp^R^)	This study
BL21 (DE3)/pEtDuet-1-*kshC*	BL21 (DE3) containing pEtDuet-1-*kshC* (Amp^R^)	This study
**Plasmids**		
pET-28a(+)	Expression plasmid, Km^R^, T7 promoter	This lab
pET-28a(+)-*kshA*	pET-28a(+) containing *kshA*	This study
pET-28a(+)-*kshB*	pET-28a(+) containing *ksbB*-His	This study
pET-28a(+)-*kshC*	pET-28a(+) containing *kshC*	This study
pET-28a(+)-*fdh*	pET-28a(+) containing *fdh*	This lab
pEtDuet-1	Expression plasmid, Amp^R^, T7 promoter	This lab
pEtDuet-1-*kshC*	pEtDuet-1 containing *kshC*-His	This study
**Primers (5′-3′)**		
P1	CGGGATCCATGACGACTGAGCACGCCGG	
P2	CCCAAGCTTTCAGCTTGATTGAGCGGTTTC	
P3	CGGGATCCATGACTGATGAACCGTTAGGTAG	
P4	CCCAAGCTTTCACTCGTCGTAGGTCACCTC	
P5	CGGGATCCGATGGCCGGTCTGAACAACGATAG	
P6	CCCAAGCTTTCAGCCGCTGGCCGGGGCGGCC	
P7	CGGGATCCGATGGCCGGTCTGAACAACGATAG	

Amp^R^, ampicillin-resistant; Km^R^, kanamycin-resistant, The restriction enzyme sites are underlined.

## Supplementary Materials

Supplementary Materials are available online. Supplementary File 1: Figure S1. Identification of the recombinant plasmids pET-28a-*kshA*, pET-28a-*kshB* and pET-28a-*kshC*. Supplementary File 2: Figure S2. Effect of 9-OH-AD on the activity of KSH. Supplementary File 3: Figure S3. HPLC result of AD and 9-OD-AD. (a) Standard sample of 9-OH-AD; (b) Standard sample of AD; (c) Conversion results at 0 h; (d) Conversion results at 6 h. Supplementary File 4: Figure S4. NMR result of 9-OH-AD. Supplementary File 5: Sequences of codon optimized *kshA*, *kshB,* and *kshC.*
