# Supplementary material for: A Novel 3-Phytosterone-9α-Hydroxylase Oxygenation Component and Its Application in Bioconversion of 4-Androstene-3,17-Dione to 9α-Hydroxy-4-Androstene-3,17-Dione Coupling with A NADH Regeneration Formate Dehydrogenase
Source: Molecules. 2019 Jul 11;24(14):2534. doi: 10.3390/molecules24142534 (PMC6680482; doi:10.3390/molecules24142534)
Supplement: Supplementary file 1 [file molecules-24-02534-s001.pdf]

A novel 3-phytosterone-9α-hydroxylase oxygenation component and its application in bioconversion of 4-androstene-3,17-dione to 9α-hydroxy-4-androstene-3,17-dione coupling with a formate dehydrogenase

Xian Zhang[1], Manchi Zhu[1], Rumeng Han[1], Youxi Zhao[2], Kewei Chen[3], Kai Qian[4], Minglong Shao[1], Taowei Yang[1], Meijuan Xu[1], Jianzhong Xu[1,*], Zhiming Rao[1,*]

[1] The Key Laboratory of Industrial Biotechnology, Ministry of Education, School of Biotechnology, Jiangnan University, 1800 Liu Avenue, Wuxi, Jiangsu 214122, P.R. China

[2] Biochemical Engineering College, Beijing Union University, Beijing 100023, P.R. China

[3] School of Food Science and Technology, Jiangnan University, 1800 Liu Avenue, Wuxi, Jiangsu 214122, P.R. China

[4] School of Medicine, Yichun University, Yichun 336000, Jiangxi, P.R. China

*Correspondence: xujianzhong@jiangnan.edu.cn (J. X.); raozhm@jiangnan.edu.cn (Z. R.); Tel.: +86-1585-283-4439 (J. X.); +86-1392-113-5816 (Z. R.)

Supplementary File 1

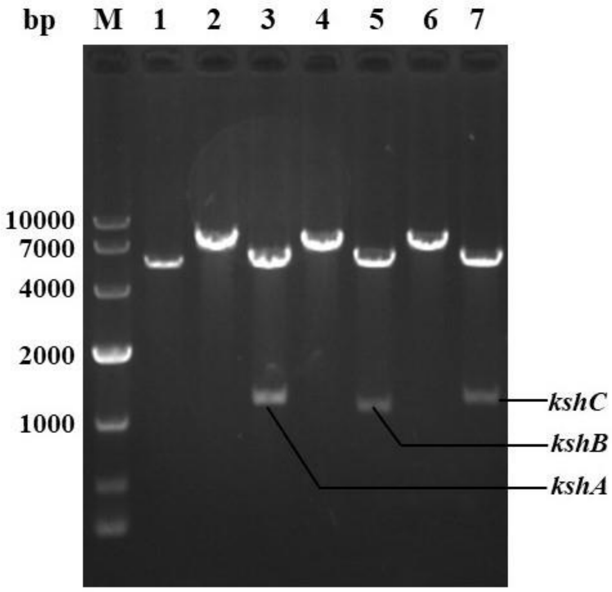

Figure S1. Identification of the recombinant plasmids pET-28a-*kshA*, pET-28a-*kshB* and pET-28a-*kshC*

M: DL 10,000 DNA Marker; 1: pET28a/*Bam*H I; 2: pET28a-*kshA*/*Bam*H I; 3: pET28a- *kshA*/*Bam*H I+*Hind* III; 4: pET28a-*kshB*/*Bam*H I; 5: pET28a-*kshB*/*Bam*H I+*Hind* III; 6: pET28a-*kshC*/*Bam*H I; 7: pET28a-*kshC*/*Bam*H I+*Hind* III

Supplementary File 2

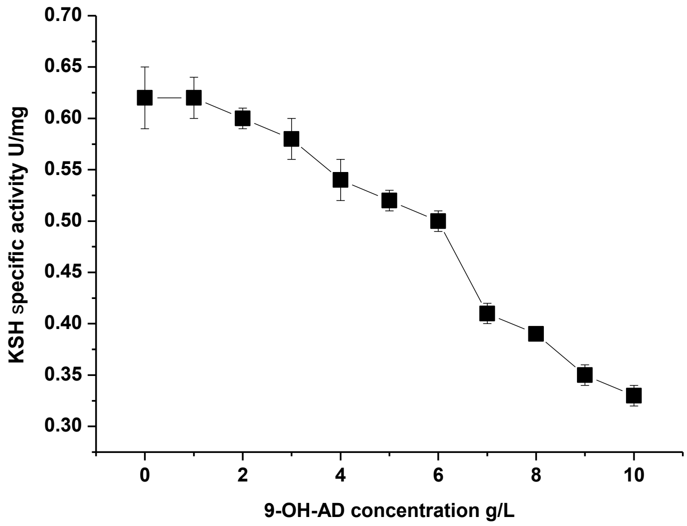

Figure S2. Effect of 9-OH-AD on the activity of KSH.

All assays were performed in triplicate, and standard deviations of the biological replicates are shown.

Supplementary File 3

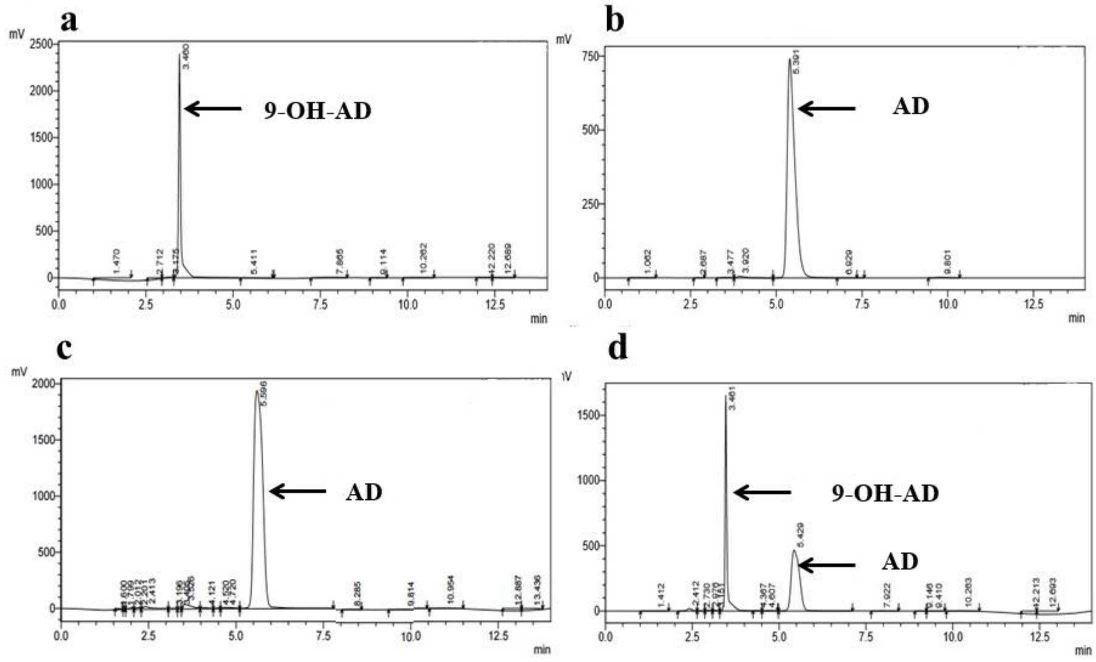

Figure S3. HPLC result of AD and 9-OD-AD. (a) Standard sample of 9-OH-AD; (b) Standard sample of AD; (c) Conversion results at 0 h; (d) Conversion results at 6 h.

Supplementary File 4

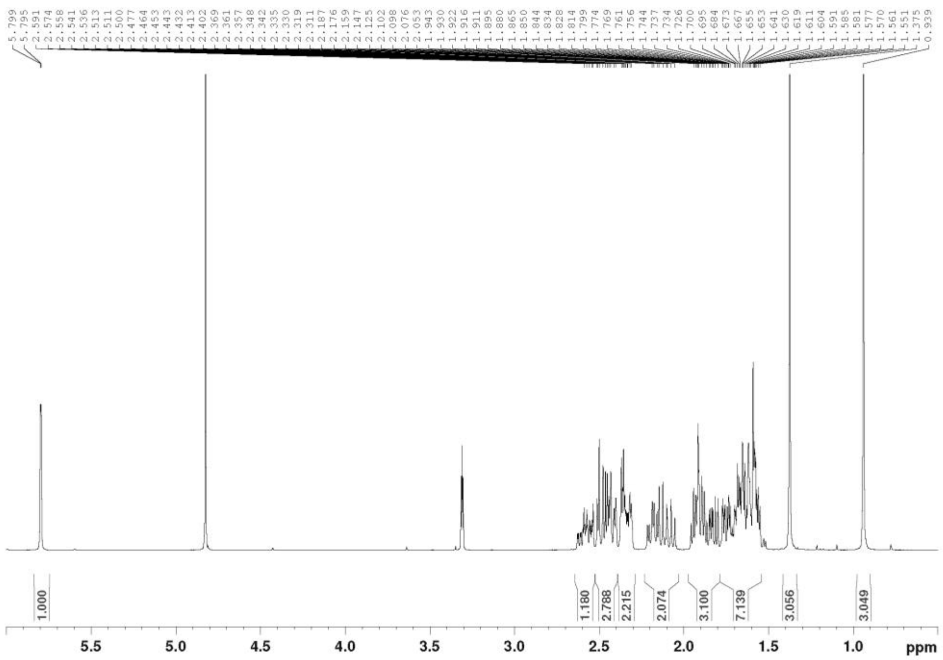

Figure S4. NMR result of 9-OH-AD.

Supplementary File 5

Sequences of codon optimized *kshA*, *kshB* and *kshC*

kshA

1 ATGACGACTG AGCACGCCGG AATCCGTGAG ATTGACACAG GCGCGTTGCC TGATAGATAC
61 GCGCGCGGAT GGCACTGCCT AGGACCGGTG AAGAACTTCT TAGATGGCCA GCCGCATAGC
121 GTTGAGATCT TCGGCACTAA GTTAGTAGTA TTCGCTGACA CTAAAGGTGA ACTTAAGATC
181 TTAGACGGCT ACTGCCGGCA CATGGGCGGT GACCTGAGCC AGGGCACGAT TAAGGGTGAT
241 GAAGTAGCAT GCCCGTTCCA TGACTGGCGC TGGGGCGGTG ACGGTAAGTG TAAGTTAGTG
301 CCGTACGCTA AGCGCACCCC TAGGTTAGCG AGGACTAGAG CGTGGCACAC TGACGTGAGA
361 GGCGGGCTGC TCTTCGTCTG GCATGACCAT GAAGGTAATG ATCCACAGCC TGAGGTGAGA
421 ATCCCTGAAA TACCTGAGGC CGCGTCTGAT GAGTGGACGG AGTGGCAGTG GAACTCCATG
481 CTGATCGAGG GCTCCAACTG CCGCGAGATC ATCGACAACG TGACCGACAT GGCCCACTTC
541 TTCTACATCC ACTTCGGCCT GCCGACCTAC TTCAAGAACG TGTTCGAGGG CCACATCGCC
601 TCGCAATACC TGCACAACGT GGGCCGCCAG GACATCGGCG GCATGGGCAC GCAGTACGGC
661 GAGAGCCACC TGGACTCCGA GGCGAGCTAC TTCGGCCCGA GCTTCATGAT CAACTGGCTG
721 CACAACAACT ACTCCGGCTA CAAGGCGGAG AGCATCCTGA TTAACTGCCA CTACCCGGTC
781 ACTCAGGACT CCTTCATGCT TCAGTGGGGC GTGATCGTCG AGAAGCCGAA GGGCATGGAC
841 GAGAAGACCA CCCAGAAGCT GGCCAACGCC ATGACGGACG GCGTCAGCCA GGGCTTCCTG
901 CAGGACGTCG AGATCTGGAA GCACAAGACG CGCATCGACA ACCCGCTGCT GGTCGAGGAA
961 GACGGCGCGG TCTACCAGAT GCGCCGCTGG TACCAGCAGT TCTACGTCGA CGTGGCCGAC
1021 ATCACCCCTG ACATGACCGA CCGCTTCGAA ATGGAGATCG ACACGACCGC GGCCAACGAA
1081 AAGTGGCACG TCGAAGTTGA GGAGAACCTG AAGATCCAGG CCGAACAGAA GGCGGCGGAG
1141 AAGGAAACCG CTCAATCAAG CTGA

kshB

1 ATGACTGATG AACCGTTAGG TAGTCACGTT TTAGAATTGC AGGTGAGCGC AGTGATCGAA
61 GAAACAGCGG ATGCTAGATC ATTGGTGTTC GCAGTGCCGG AAGGAAGTAC TATCCCTGAA
121 GATAGACTGA GATATAGCCC AGGTCAGTTC TTAACATTGA GAGTCCCGAG TGATAGAACC
181 GGCAGTGTGG CCCGATGTTA CAGCTTGAGT AGTAGTCCAG TAACCGATGA TCAACTGACG
241 GTGACAGTGA AACGCACAGC AGATGGTTAC GCAAGTAACT GGTTATGTGA TAATGCGCAC
301 GCGGGTATGA AAATGCACGT CTTAGCCCCG TCAGGTACCT TCGTCCCGAA AGATCTGGAT
361 ACGGATTTCT TACTGTTGGC CGCCGGAAGT GGCATCACAC CGATGATGGC CATCTGTAAG
421 TCAGCTTTAG CAGAAGGAAG CGGTAACGTT GTCTTAGTGT ACGCCAATAG AGATGAAAAC
481 AGCGTCATCT TCGGTGCCAC CTTGAGAGAG CTGGCGGCAA AATATCCGGA CAGATTCACC
541 GTTGTGCATT GGTTAGAGAC CGTGCAGGGC TTACCGAGTC CGGCCGCCCT GGCCGGCCTG
601 CTGGCGCCGT ACGCGAGCCG CGAGGCCTTC ATCTGCGGTC CGGGCCCGTT CATGGCCGCC
661 GCGGAGCAGG CCCTGCAGCA GGCCGGCGCG GCGGACGAGC GCATCCACAT CGAGGTCTTC
721 AAGTCGCTGG ACTCCGACCC GTTCGCCGCG GTCGTGATCG AGGAAGAAGA GGGCGACCAG
781 GAGCCGGCCA CCGCGGTCGT CACCCTGGAC GGCACCACGC ACGAGGTCCG CTGGCCGCGT
841 TCCGCCACCC TGCTGGACGT GCTGCTGGAC AAGGGCCTGG ACGCCCCGTT CTCCTGCCGC
901 GAGGGCCACT GCGGCGCGTG CGCGGTGCTG AAGAAGAGCG GCGACGTGGA GATGAAGATC
961 AACGACGTCC TGGAGCCGAG CGACCTGGAA GAGGGCCTGA TCCTGGGCTG CCAGGCCACC
1021 CCGGTGTCCG ACAGCGTCGA GGTGACCTAC GACGAGTGA

kshC

```
1       ATGGCCGGTC TGAACAACGA TAGTACAAGA GCCTCAGTCA GAGAAATCGA CGTAGGTCAA
61      TTGCCAACAC GTTTCGCGCG TGGATGGCAT TGTTTGGGTT TGGTGAGTGA CTTCGTAGAC
121     GGTCAGCCGC ATTCAATCAC AGCTTTCGGC ACCAAACTGG TAGTCTTTGC CGATAGCCAT
181     GGTAGTGTCC ATGTTCTGGA TGCCTATTGT CGACACCTGG GTGGTGACTT AAGTCAGGGT
241     AAAGTCAAAG GTGACGCCGT CGCGTGCCCG TTCCATGATT GGCGTTGGGC GGGAAACGGT
301     AGATGCGCCC AGGTCCCGTA CGCCAAGAGA GCACCTAGAT TAGCGAGAAC GAGAGTCTGG
361     AGAACCAGTG TGCGTTCAGG TCTGCTGTTC GTCTGGCATG ATCCGGAAGG TAGTGTGCCG
421     AGTCCGCATC TTGATATCCC AGATATCCCG GAAGTTAGGG ACCCTGGTTG GACCGAGTGG
481     AGTTGGAGAA GTGAATTAAT CGGTAGCAAC TGTAGAGAAA TCGTTGACAA CATCGTGGAT
541     ATGGCCCATT TCTATTACAT CCATTTCGGT TTCCCGACCT ATTTCAAAAA TGTGTTCGAA
601     GGTCAAGTGG CGTCTCAGTA TCTGCGTACC ATCGGTCGCC CGGATGTCCA CCTGGGCGGC
661     TCGCACTACG CCGGCGAGCA GGTGCTGGAC AGCGAGGCGA GCTATTTCGG CCCGTCCTTC
721     ATGATCAACC GCCTGCACAA CAGCTACAGC GGCTACGAGG TCGAGGCCAT CCTGGTGAAC
781     TGCCACTACC CGGTCACCCC GGAGAGCTTC GTCCTGCAGT GGGGCATCAT GGTGCGCCGC
841     CCGCAGGGCT TGTCAGAAGA GGCCACCGAC CGTCTGCTGC ACGCCTTCAC GGAGGGCGTG
901     TCCTCCGGCT TCCTGCAGGA CGTGGAGATC TGGAAGAACA AGACGCGCAT CGACAACCCG
961     CTGCTGGTCG AGGAAGACGG CCCGGTCTAC CAGCTGCGCC GCTGGTACGA ACAGTTCTAC
1021    GTCGACGCCG CCGACGTGAC CCCGGAGATG ACGGACCGCT TCGAGTACGA GGTCGACACG
1081    ACCGCCGCCA ACGAGTACTG GCGCTCCGAG GTCGCCGAGA ACCTGCGCCA GCGCGGCGCC
1141    GCGGCCGCCC CGGCCAGCGG CTGA
```
